# TcMEP threshold change is superior to A-train detection when predicting facial nerve outcome in CPA tumour surgery

**DOI:** 10.1007/s00701-020-04275-z

**Published:** 2020-03-07

**Authors:** Tom Hendriks, Henricus P. M. Kunst, Maarten Huppelschoten, Jonne Doorduin, Mark Ter Laan

**Affiliations:** 1 Department of Neurosurgery, Radboud University Medical Center, Nijmegen, The Netherlands; 2 Department of Otorhinolaryngology, Radboud University Medical Center, Nijmegen, The Netherlands; 3 Department of Otorhinolaryngology, Medical Center, Maastricht, Netherlands; 4 Department of Neurology, Donders Institute for Brain, Cognition and Behaviour, Radboud University Medical Center, Nijmegen, The Netherlands

**Keywords:** Facial nerve, Cerebellopontine angle tumours, Intraoperative neuromonitoring, Motor evoked potentials, Electromyography

## Abstract

**Object:**

Surgery of tumours in the cerebellopontine angle (CPA) can lead to loss of facial nerve function. Different methods of intra-operative nerve monitoring (IOM) (including free-running EMG, direct nerve stimulation and transcranial motor evoked potentials (TcMEP)) have been used to predict facial nerve outcome during surgery. Recent research has shown TcMEP threshold increase and the occurrence of A-trains on the EMG to have great potential in doing so. This study compares these two methods and correlates them to House-Brackmann (HB) scores post-op in patients with tumours in the cerebellopontine angle.

**Method:**

Forty-three patients (one was operated twice) with large CPA tumours treated surgically in the Radboud University Medical Center between 2015 and 2019 were included in this study. During surgery, TcMEP threshold increases and A-train activity were measured. Because our treatment paradigm aims at facial nerve preservation (accepting residual tumour), TcMEP threshold increase of over 20 mA or occurrence of A-trains were considered as warning signs and used as a guide for terminating surgery. HB scores were measured post-op, at 6 weeks, 6 months and 1 year after surgery. Spearman’s correlation was calculated between the IOM-values and the HB scores for a homogeneous subgroup of 30 patients with vestibular schwannoma (VS) without neurofibromatosis type II (NF-II) and all patients collectively.

**Results:**

TcMEP threshold was successfully measured in 39 (90.7%) procedures. In the homogeneous VS non-NFII group, we found a statistically significant moderate-to-strong correlation between TcMEP threshold increase and House Brackmann score immediately post-op, at 6 weeks, 6 months and 1 year after surgery (Spearman’s rho of 0.79 (*p* < 0.001), 0.74 (*p* < 0.001), 0.64 (*p* < 0.001) and 0.58 (*p* = 0.002), respectively). For A-trains, no correlation was found. Similar results were found when including all patients with CPA tumours. A threshold increase of < 20 mA was a predictor of good facial nerve outcome.

**Conclusion:**

These results show that TcMEP threshold increases are strongly correlated to post-operative HB scores, while A-trains are not. This suggests TcMEP threshold increases can be a valuable predictor for facial nerve outcome in patients with large tumours when facial nerve preservation is prioritized over total resection. In this study, we found no use for A-trains to prevent facial nerve deficits.

## Introduction

An important complication after surgical resection of cerebellopontine angle (CPA) tumours is the loss of facial nerve (FN) function [3, 16]. In order to prevent this complication, monitoring of the facial nerve during surgery (intraoperative neuromonitoring, IOM) is applied. The most used intra-operative techniques include identification of the facial nerve with direct nerve stimulation (DNS), visual detection of A-trains using free-running electromyography (EMG) and measuring transcranial motor evoked potentials (TcMEP) of the facial nerve [2].

Previous research has shown potential for using A-trains as a predictor for post-operative facial nerve function [13, 15], as well as for using different forms of TcMEP analyses [1, 22]. Superiority of either one is yet to be determined, but promising results using TcMEP threshold increase and TcMEP amplitude ratios have been demonstrated [6, 11, 17].

**Fig. 1:**
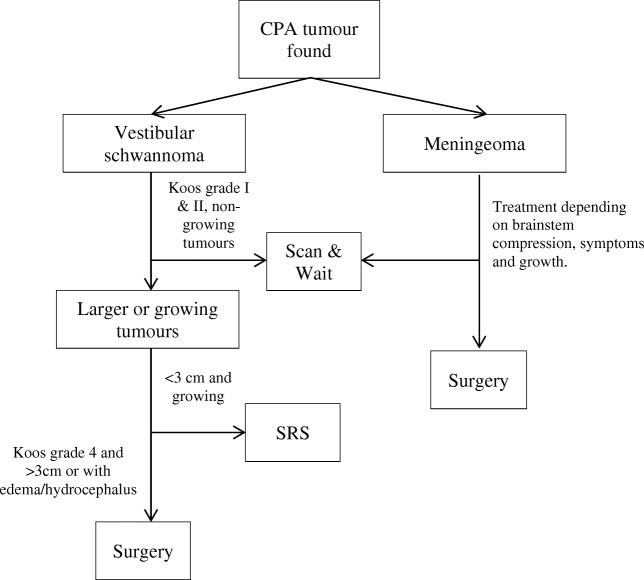
Flowchart showing the treatment algorithm used in the Radboud University Medical Center for the treatment of (common) CPA tumours

Within the Radboud University Medical Center’s treatment algorithm for suspected vestibular schwannomas, small tumours are monitored for growth using a ‘wait and scan’ approach. When growth is shown, tumours smaller than 3 cm diameter without edema or hydrocephalus are treated using stereotactic radiosurgery. Surgery is only performed on Koos grade 4 tumours (indicating brainstem compression) larger than 3 cm or with significant edema or hydrocephalus. Suspected meningeomas are operated upon depending on compression, symptoms and growth (Fig. 1).

During surgery, multimodal IOM, including free-running EMG, TcMEP and DNS, was used as a guide for stopping surgery. The main surgical goal consists of the decompression of the brainstem and cranial nerves while maintaining facial nerve function.

The goal of the current study is to determine which IOM modality correlates better with post-operative facial nerve function and finding a reliable intra-operative parameter to be used as a guidance for preserving maximal facial nerve function, which has thus far not been found.

## Methods

Data was recorded prospectively on 43 procedures performed on 42 patients with a CPA tumour between 2015 and 2019. All patients presenting with CPA tumours that required surgery at the Radboud University Medical Center were included. The final follow-up data was collected on December 3, 2019. All procedures were performed using multimodal IOM. Baseline characteristics consisting of age, sex, presence of neurofibromatosis type II mutation and location (left/right) of the tumour were recorded pre-operatively. The type of tumour was suggested radiologically pre-operative, but finalized by pathology post-op. IOM and clinical symptoms were recorded pre-operatively and during follow-up.

Surgery was performed through a standard retrosigmoid approach, without retractors in supine positon with the head turned and tilted towards the contralateral side. Total intravenous anaesthesia was administered using propofol and opioids. During surgery the facial nerve was monitored using free-running EMG, TcMEP and DNS. To do this, needle electrodes were placed in the mm. orbicularis oculi, orbicularis oris, nasalis and mentalis. The trigeminal nerve was also monitored using a needle electrode in the m. masseter. When hearing was (partly) intact, brainstem auditory evoked potential (BEAP) was measured during surgery, although hearing preservation was not a surgical goal. For TcMEP, we used the threshold-level method as described by Sarnthein in 2013 [17]. In short, transcranial electrical stimulation was delivered through corkscrew electrodes at C3/C4 versus Cz. We used rectangular biphasic pulses with an anodal phase of typically 0.4 m applied in trains of 5 pulses with an interpulse interval of 1 ms. TcMEP stimulation baselines were set before incision and checked after dural opening. Baseline TcMEP threshold was determined in steps of 5 mA until a MEP was detected in one of the target muscles of the facial nerve. A MEP response as low as 50 μV with appropriate response latency qualified as a reliable MEP response. The testing was repeated every 30 s. Threshold increase of more than 20 mA to reproduce baseline MEP or occurrence of A-trains were considered as warning signs. A-trains were defined as described previously [13]. DNS was used for intraoperative identification of the facial nerve using a monopolar probe. Before resection, the tumour capsule was stimulated to confirm whether there were any facial nerve fibres in the area. Thereafter, DNS was frequently used throughout the surgical procedure to identify and/or confirm the trajectory of the facial nerve. Stimulation intensity varied between 0.05 and 0.5 mA with an anodal pulse of 200 μs. Depending on the recovery of TcMEP, the durations of the A-trains and anatomical view of the facial nerve, surgery was either continued or terminated.

Typical follow-up consisted of a consultation at around 6 weeks post-operatively, around 6 months, and then yearly. MRI scans were made at 3 months and then yearly.

Correlations between House-Brackmann grade score and IOM data was determined by calculating Spearman’s correlation coefficient using IBM SPSS Statistics 24.

## Results

### Patient and tumour characteristics

The average age of the patients at time of surgery was 52 (15–77) years old. Twenty-three out of 43 patients (53.5%) were male. Two (4.7%) patients were diagnosed with neurofibromatosis type II. Post-operative pathology showed 34 (79.1%) schwannomas, 5 (11.6%) meningiomas, 2 (4.7%) ependymoma, 1 (2.3%) plexus papilloma and 1 (2.3%) epidermoid cyst (Table 1). Of the 35 CPA tumours, the average volume was 14.5 (2.1–32) cm^3^. The average pre-operative tumour diameter was 35 (19–53) mm. Twenty-three were right-sided (53.5%).

**Table 1:** Patient and tumour characteristics. Age is shown rounded to 1 decimal, the rest in absolute numbers (range or percentage)

Patient and tumour characteristics	*n* = 43
Average age (range)	52 years (15–77)
Male	23 (53.5%)
Neurofibromatosis type II	2 (4.7%)
Schwannoma	34 (79.1%)
Meningioma	5 (11.6%)
Other	4 (9.3%)
Right sided	23 (53.5%)
Average tumour size in mm	35.0 (19–53)
Average tumour size in cm^3^	14.5 (2.1–32)

### Surgical results

Average surgery time was 283 (150–477) minutes. The median length of stay was 6 days (4–21), including 1 day prior to surgery and the day of surgery.

When taking into account only tumours in non-fibromatosis patients with VS (*n* = 30), the results were as follows. The average pre-operative schwannoma size was 15.7 (4.84–32) cm^3^. The average pre-operative tumour diameter was 36.4 (19–53) mm. Fifteen were right-sided (50%). Average surgery time was 277 (168–476) minutes. Near total resection (defined as a rest of <1 cm^3^) was achieved in 71.4% (20 out of 28 patients (excluding 2 patients without a post-op scan)). Median tumour rest was 0.59 cm^3^ (0–7.9). The median length of stay was 6 days (4–21), including 1 day prior to surgery and the day of surgery (Table 2).

**Table 2:** Surgical results of all patients and of non-NF II vestibular schwannoma patients. Data are represented as absolute values (range, percentage)

Surgical results	All tumours (*n* = 43)	Vestibular schwannoma non-NF II (*n* = 30)
Average surgery time in minutes	283 (150–477)	277(168–476)
Median rest in cm^3^	0.57 (0–10.5)^a^	0.59 (0–7.9)^b^
Near total resection	30 (68.8%)	20 (71.4%)
Median LOS in days	6(4–21)	6 (4–21)

^a^*n* = 41

^b^*n* = 28

Post-operative complications consisted of decrease in NVII function directly post-op, hearing loss, trigeminal issues, balance/cerebellar issues, minor dysphagia, hydrocephalus and CSF leakage (Table 3).

**Table 3:** Complications recorded post-op and during follow-up shown for all patients and the VS non-NF-II subgroup. Values are absolute numbers (percentage)

Complications	All patients (*n* = 43)	Vestibular schwannoma non-NF II (*n* = 30)
Decrease in NVII function (immediately post-op)	21 (48.8%)	16 (53.3%)
Trigeminal issues	4 (9.3%)	4 (13.3%)
Balance/cerebellar issues	4 (9.3%)	3 (10.0%)
Minor dysphagia	4 (9.3%)	1 (3.3%)
Bleeding	0	0
Hydrocephalus	1 (2.3%)	1 (3.3%)
CSF leakage	1 (2.3%)	1 (3.3%)
Hearing loss^a^	2 out of 4 (50%)	1 out of 1 (100%)

^a^All other patients were deaf before start of surgery

In 1 (2.3%) patient where resection was stopped because of TcMEP threshold increase and multiple A-trains, leaving a considerable rest, tumour regrowth occurred after 2 years.

### Facial nerve outcomes and follow-up

**Fig. 2:**
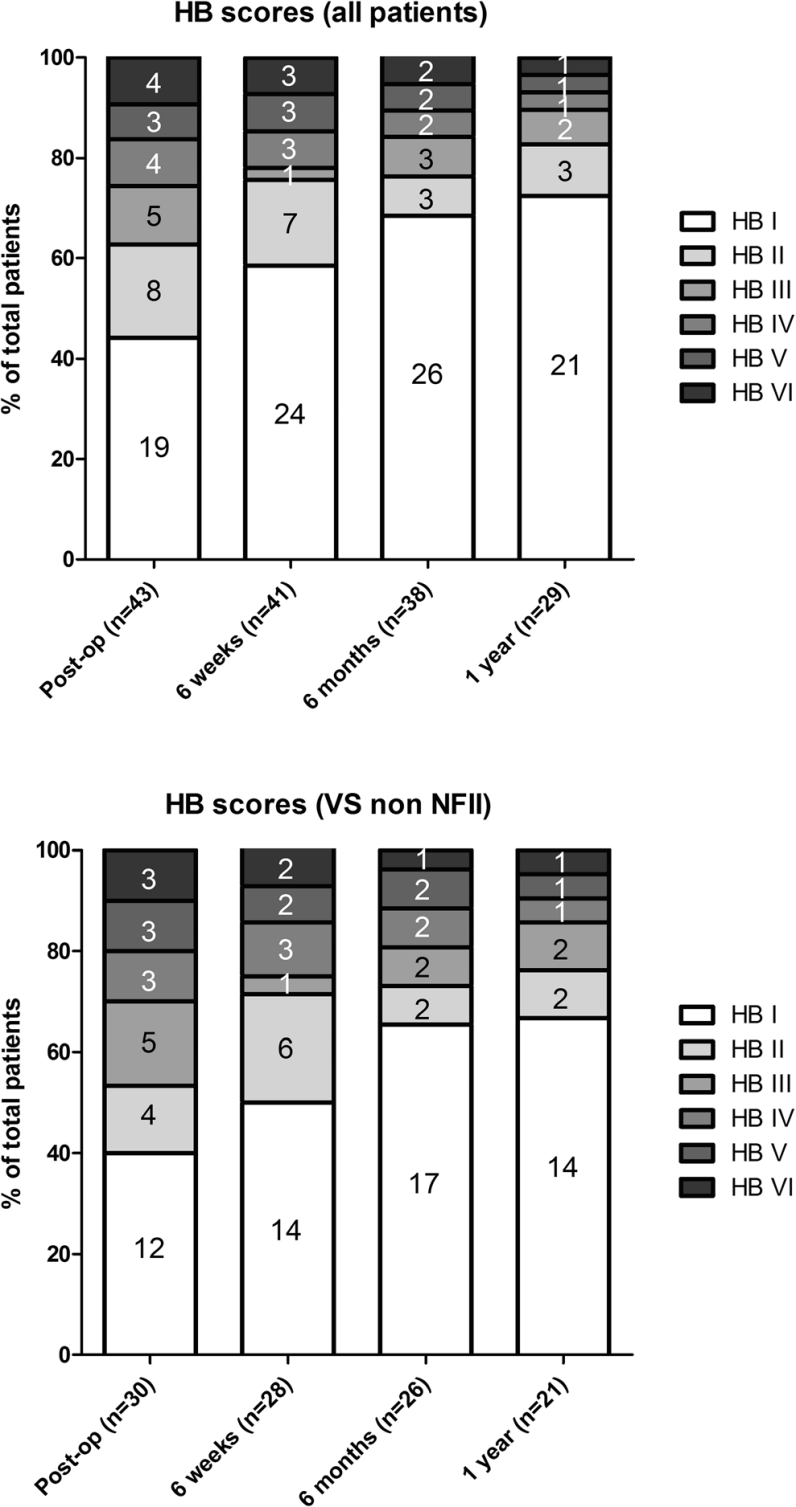
Bar chart of House-Brackmann scores shown at different times post-op for all patients and separately for VS non-NF-II patients. Data are shown in absolute values

In the total group, post-operative House-Brackmann scores were recorded in 43 patients post-op, in 41 patients at 6 weeks, in 38 patients at 6 months and in 29 patients at 1 year (Fig. 2).

At 6 months, 76% of all patients had good facial nerve outcome (HB 1 or 2); for non-NF vestibular schwannoma patients this was 73%. At 1 year, this was 83 and 76% respectively.

### IOM results

TcMEP threshold was successfully measured in 39 (90.7%) procedures. In 7 (16.3%) cases a sudden loss of TcMEP occurred, even with highly increased stimulation. In these cases a threshold increase of 100 mA was defined to enable correlation analysis. In 4 (9.3%) cases the TcMEP was not reliably measurable at the start or during surgery. A-trains were obtained in 37 (86.0%) of cases.

### Correlation between IOM measurements and HB score

**Fig. 3:**
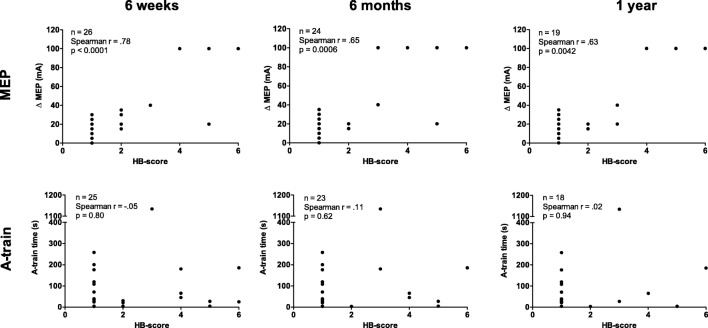
Graphs showing correlation between MEP threshold change (∆MEP) and A-train time at 6 weeks and at 6 months follow-up in the VS non-NF-II group. X-axis represents House-Brackmann score for facial nerve function. Rho: Spearman’s correlation coefficient

A graphical presentation of IOM data vs. HB score outcomes at different follow-up intervals can be seen in Fig. 3. In the homogeneous VS non-NFII group, we found a statistically significant moderate-to-strong correlation between TcMEP threshold increase and House-Brackmann score immediately post-op, at 6 weeks, at 6 months and at 1 year (Spearmans rho of 0.80 (*p* < 0.001), 0.78 (*p* < 0.001), 0.65 (*p* = 0.001) and 0.63 (*p* = 0.004), respectively). For A-trains, no correlation was found (Spearman’s rho of − 0.06 (*p* = 0.750), − 0.05 (*p* = 0.798), 0.11 (*p* = 0.623) and 0.04 (*p* = 0.867), respectively). (Excluding the outlier (1134s A-train) does not result in a better correlation.)

When including all patients, correlation coefficient results were comparable (Spearman’s rho of 0.79 (*p* < 0.001), 0.74 (*p* < 0.001), 0.64 (*p* < 0.001) and 0.58 (*p* = 0.002), respectively). For A-trains, no correlation was found (Spearman’s rho of 0.07 (*p* = 0.727), 0.05 (*p* = 0.785), 0.16 (*p* = 0.398), and 0.09 (*p* = 0.693), respectively).

Out of 18 patients in the VS non-NF II group that had a TcMEP threshold change of less than 40 mA and a follow-up at 6 months, 17 (94%) had good (HB grade I or II) facial nerve outcome at 6 months post-operatively. All 9 patients in this group with a threshold change of less than 20 mA had good facial nerve outcome.

## Discussion

We have found that all patients with schwannoma and a TcMEP threshold change of less than 20 mA have good facial nerve outcome. Also, no correlation was found between A-train time and facial nerve outcome.

Overall good outcome was achieved in our series as for facial nerve function (76% HB < 3 at 6 months), and complication rates were low. The post-op facial nerve function loss recovered quickly in most patients. We have found a moderate (overall population) to strong (considering only non-NF vestibular schwannoma) correlation between the TcMEP threshold change and functional facial nerve outcome in a mixed population of large CPA tumours. Our surgical results in vestibular schwannoma patients in terms of facial nerve function and complications are comparable/superior to previous reports on resection of large vestibular schwannomas [3, 6–10, 12, 18, 19, 21, 23–25].

The TcMEP threshold we have found is in agreement with the findings by Sarnthein [17]. In addition to this, our findings also show that in 94% of patients with a threshold change of less than 40 mA, good facial nerve function was preserved. Our and Sarnthein’s study used a neuromonitoring device with constant-current transcranial electric stimulation (measured in amperes), while other devices on the market use constant-voltage transcranial electric stimulation (measured in volts). Both stimulation methods can be used to elicit MEP responses. Studies using constant-voltage stimulation, but with a similar threshold-level method for TcMEP of the facial nerve, found that a high delta MEP (57–70 V) is correlated with long-term post-operative facial nerve damage [5, 20]. These results support our findings, but the exact threshold cannot be compared due to the different stimulation protocol. Using a different TcMEP technique, Matthies et al. reported *a* > 55% pre- and post-op TcMEP amplitude ratio as a predictor for good long-term facial nerve function [11]. Similarly, Bhimrao et al. reports the good facial nerve function for a 60% pre- and post-op amplitude ratio [4].

Duarte et al. in 2015 used direct nerve stimulation and concluded proximal amplitudes to possibly predict facial nerve function [6]. They also looked at different techniques for predicting nerve function, eventually suggesting a compound score might be necessary, because no single parameter is a sufficiently good predictor for facial nerve outcome.

Our results indicate that TcMEP threshold increase is sufficient as a single parameter to predict facial nerve outcome. This method, besides showing good correlation to facial nerve outcome, has several advantages over using TcMEP amplitude ratios (as described by Matthies) [11] and A-trains. The TcMEP threshold method can be applied using lower stimulus intensity. This reduced muscle twitching during surgery. Furthermore, threshold decreases are easier to quantify. Instead of measuring just the maximal amplitude, the observer compares the full polyphasic response to the previous one. This removes part of the variability normally seen in amplitude ratios. An important difference between TcMEP and A-train time is that TcMEP thresholds are recorded every 30 s during surgery, while A-train time is calculated in a post-op analysis. This means TcMEP threshold increase is known by the surgeon during surgery, while for A-trains it is only known *if* they occur, not the total amount of train time.

In our series, no relation was found between A-train time and facial nerve outcome, which is surprising, considering the results previously published [15]. A systematic review describes the history of free-running EMG as a predictor for facial nerve outcome [2]. The predictive value of A-trains for long-term outcome has been a point of discussion for longer time, but some promising results were achieved. A previous study by Prell et al. showed < 0.5 s of train time to be correlated with good facial nerve outcome, whereas > 10 s was correlated with long-term facial nerve dysfunction [13]. These train times are much shorter than the ones found in our patients. Most patients in this study (including the ones with good facial nerve outcome) had train times far exceeding 10 s. This is most likely caused by the algorithm used for A-train detection. As described later by Prell, only 1.46% of A-trains are detected using the automated algorithm, while our study also includes A-trains found by visual analysis [14].

Contradictory to previous research, there are cases in our cohort with high train times and no nerve function loss, and vice versa. Difference in cohort size or the surgical goals (radical resection was not a goal in this study) might play a role in explaining these differences between our results and previous ones.

In the current study, and in our clinical practice, we use DNS for intraoperative facial nerve identification and not for function prediction. We are aware that in many studies DNS has been used to predict facial nerve outcome after tumour removal using different techniques (absolute amplitude, stimulation threshold, proximal-to-distal ratio). An extensive review has been written on this topic (Acioly 2013 World Neurosurg). Although these techniques may facilitate post-operative clinical management, they cannot be used intraoperatively to predict facial nerve outcome, because stimulation distal to facial nerve injury evokes normal responses and because the proximal part of the facial nerve is mostly not visible until complete removal of the tumour.

The strengths of our study are the relatively large cohort of subjects with large tumours, the use of multi-modal monitoring and comparison of these in a single population. Moreover, since we studied our IOM regimen in different tumours in the CPA, our results apply to not only schwannoma.

A possible limitation of our study is the fact that the surgeon was not blinded for intra-operative measurements and thus was guided by A-trains as well as TcMEP threshold change. This results in a bias towards less aggressive resections. As a result, the distribution of post-op HB grades is skewed towards the lower grades. Also, TcMEP changes seem to occur earlier than A-trains, possibly explaining the lack of correlation between A-train duration and TcMEP in our series (with continued resection, more A-trains and possibly more unfavourable facial nerve outcomes could have been found). Although commonly used, the use of the HB grade for facial nerve outcome is quite crude; unfortunately, more detailed outcome measurements (e.g. Sunnybrook classification) were not available.

Based on our results, we suggest using TcMEP threshold as a guide for safe resection of tumours in the CPA. Especially considering vestibular schwannoma, in a time where radiosurgery offers good treatment for most tumours and small remnants have shown to have low recurrent rates, the most important goal of surgery must be to preserve function. TcMEP threshold is a better predictor than A-trains when operating on large vestibular schwannomas when a safe (partial) resection is to be achieved, resulting in 71% near total resection and 73% good facial nerve function after 6 months.

## Data Availability

Part of the data has been previously shown at the European Skull Base Society Congress 2018 in Warsaw.
